# Examining human-AI interaction in real-world healthcare beyond the laboratory

**DOI:** 10.1038/s41746-025-01559-5

**Published:** 2025-03-19

**Authors:** Magdalena Katharina Wekenborg, Stephen Gilbert, Jakob Nikolas Kather

**Affiliations:** 1 Else Kroener Fresenius Center for Digital Health, Faculty of Medicine and University Hospital Carl Gustav Carus, TUD Dresden University of Technology, Dresden, Germany; 2 Department of Medicine I, Faculty of Medicine and University Hospital Carl Gustav Carus, TUD Dresden University of Technology, Dresden, Germany; 3 Medical Oncology, National Center for Tumor Diseases (NCT), University Hospital Heidelberg, Heidelberg, Germany

**Keywords:** Computational biology and bioinformatics, Health care, Health occupations

## Abstract

Artificial Intelligence (AI) is revolutionizing healthcare, but its true impact depends on seamless human interaction. While most research focuses on technical metrics, we lack frameworks to measure the compatibility or synergy of real-world human-AI interactions in healthcare settings. We propose a multimodal toolkit combining ecological momentary assessment, quantitative observations, and baseline measurements to optimize AI implementation.

## AI is transforming healthcare

Artificial intelligence (AI) is transforming healthcare through its numerous applications, including disease diagnosis, subtyping, and prognosis, as well as decision-support tools, automation, and AI-driven administrative tasks^1^. Technical advances in large language models (LLMs) have further fueled progress, enabling the application of chatbots and reasoning engines to healthcare^2^. Many AI-based tools are already used in clinical practice, such as image analysis tools for radiology or pathology^3,4^. These AI tools can automate tedious work, thereby freeing up human time for more meaningful tasks, for example, by replacing the second reader in a mammography examination^5^. In addition, AI tools can go beyond what a human can do, for example, by extracting quantitative information from routine clinical data and predicting treatment response to specific medications^6,7^. Currently, the landscape of AI is evolving even further towards models with even broader, generalist capabilities^8,9^ as well as increasingly autonomous agents^10^. These AI models are not just a piece of software that is steered by a human for a simple task, but they can execute complex chains of tasks and guide their own process, much like a human co-worker, or as Zou and Topol have recently posited, a new teammate^11^. The pace of development of medical AI is very fast, but in the near future, all medical AI tools have one thing in common: The aim of these AI applications is to interact with human users in hybrid human-AI workflows. It is in the interaction with human healthcare providers that the value of medical AI systems is realized.

**Fig. 1:**
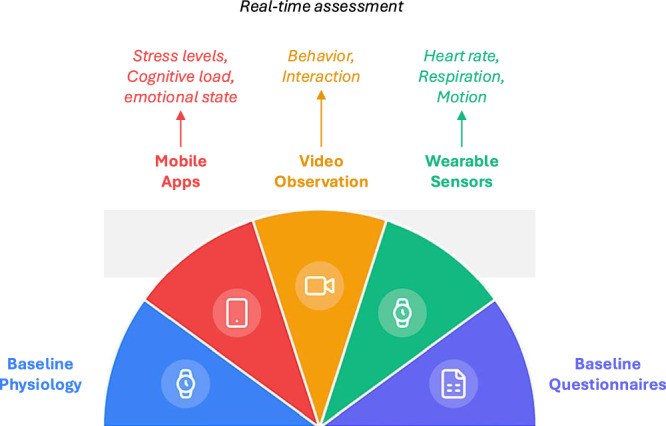
Real-world assessment of human-AI interaction in healthcare. The toolkit combines real-time and non-real-time methods to comprehensively evaluate healthcare providers’ interactions with AI systems. Real-time assessment captures immediate self-reported experiences, behavioral patterns, and physiological responses during actual AI system use in clinical settings. Non-real-time methods establish baseline characteristics through standardized physiological measurements and detailed questionnaires on professional background, digital competence, AI-related attitudes, acceptance, and adherence. Together, these complementary approaches enable quantitative evaluation of human-AI interaction, and therefore optimization of AI implementation in healthcare.

## The value of medical AI lies in human interaction

However, formal conceptual frameworks for assessing the quality of these human-AI interactions using subjective and physiological measures from a user perspective are lacking. While promising approaches exist for assessing patient user experience^12,13^, they cannot be directly applied to healthcare professionals in real-world settings. The complexity of clinical workflows and interactions with AI systems demands a clear concept of how to measure human-AI interaction in the real world.

What we cannot measure, we cannot optimize—therefore, by measuring human-AI interaction quantitatively, we can ultimately improve it. This was emphasized in a recent review by Khan et al.^14^. Most research focuses on solely technical metrics of AI performance such as sensitivity or specificity in diagnostic tasks. However, this neglects important metrics of human-AI interaction, particularly human task performance and user experience. Task performance is an objective measure of the efficiency, effectiveness, and accuracy with which a user completes a task using an AI system. User experience—following a definition in the ISO 9241-210 standard—describes a user’s holistic experience of the interaction including person’s perceptions and responses resulting from the use or anticipated use of an AI system. Understood this way, user experience goes beyond usability. The latter focuses on the subjective instrumental evaluation of how effectively, efficiently, and satisfactorily goals are achieved. While usability is a necessary component of user experience, it represents only one part of the broader, more holistic experience (Turner, 2017).

Previous work has touched upon this topic but has not provided comprehensive solutions. In a recent seminal work by Vaccaro et al., “*When Combinations of Humans and AI Are Useful: A Systematic Review and Meta-Analysis*,” the authors provided a comprehensive overview of task performance in human-AI interactions across various domains, including healthcare^15^. Similarly, other studies examined user experience of human-AI interaction, across domains and within healthcare^16^. However, there is an important limitation to the vast majority of the existing evidence: It measures human-AI interaction in laboratory settings, but not in real-world environments. Why is this a problem? In healthcare, the realities of clinical practice—such as workload (time pressures, distractions, ergonomic constraints), institutional standards (disease variability, clinical workflows, institutional ideologies), and economic constraints —differ substantially from controlled laboratory conditions. Findings from laboratory studies often fail to translate reliably to clinical workflows. This calls into question whether there exists a formalized, quantitative understanding of human-AI interaction in healthcare. We need to measure task performance and user experience in the real world, not in simulated environments. This is especially important because task performance and user experience are interlinked^17^: For example, poorly designed systems reduce both user satisfaction and efficiency, as seen with electronic health record (EHR) systems. Here, central aspects of user experience, namely usability and satisfaction, have been shown to influence the adoption of efficiency strategies among physicians^18^. While a variety of AI applications like large language models (LLMs) for clinical summarization^19^ may offer smoother integration and fewer disruptions compared to the transition from paper charts to EHRs, it remains essential to evaluate their impact in real-world clinical practice rather than simulated environments. As with many previous technologies, we cannot rule out that a technology which is well-behaved in the laboratory has unintended consequences and causes user frustration in the real-world setting.

## How to measure human-AI interaction in the real world

A number of studies have put forth formalized quantitative toolkits to measure task performance in human-AI interactions in medicine^20^. Indeed, task performance is usually the main and only aim of many AI studies in healthcare. Common metrics of task performance include diagnostic performance measures (e.g., sensitivity, specificity, area under the curve), task completion metrics (e.g., task completion time, success rate, error rate), clinical workflow efficiency (e.g., patient throughput or time spent on documentation before and after AI integration), and safety and error reduction metrics (e.g., number of adverse events avoided or improvements in patient outcomes). There are ongoing debates about how to evaluate specific aspects of task performance, such as whether to focus on human augmentation (where the human-AI system outperforms humans alone) or human AI synergy (where the system performs worse than at least one of its components, either the human or the AI alone) as pointed out by Vaccaro et al. ^15^. Most metrics either already represent core clinical practice measures, such as patient outcomes and workflow efficiency, or can be easily transferred from laboratory settings to real-world applications. Measures like task completion time, success rate, and error rate remain consistent across both contexts, making their applicability in clinical practice relatively straightforward.

In contrast, adapting methods for assessing user experience from laboratory studies to real-world healthcare settings is far more challenging due to the complexity and variability of clinical environments. This is likely one of the main reasons why efforts to measure user experience in real-world healthcare have not kept pace with AI developments. From our perspective, the challenge is not to decide what should be measured in terms of user experience. Key components of user experience are well established and validated through previous work, each highlighting different aspects of user experience. Hassenzahl et al.^21^ pointed out that user experience is shaped by the balance between pragmatic quality (usability and functionality) and hedonic quality (pleasure and identity formation). Robert and Lesage^22^ emphasized, besides the introduction of additional dimensions of user experience, the importance of both anticipated and ongoing user experience for shaping the overall experience. The Components of User Experience (CUE) model by Thüring and Mahlke^23^ identifies three distinct dimensions, namely task-related and non-task-related qualities, and emotion, and stresses the role of the system, the user, and the context of interaction. In our view, the real issue lies in determining how these aspects can be measured in real-world healthcare settings in a pragmatic and doable way. While some of these approaches mentioned above provide concrete guidance on how to measure user experience, a comprehensive toolkit that captures all user experience relevant aspects in real-world settings is still lacking.

We argue that well-established and validated behavioral and social science tools can be utilized to evaluate user experience across all types of human-AI interaction in healthcare. The insights gained from this evaluation can then be used for the development of structured and standardized processes for designing and improving future health AI, ultimately optimizing AI’s real-world value. Surprisingly, this area of research remains largely underexplored.

To address the existing gaps in understanding user experience during human-AI interactions in the real world of healthcare, we propose a multimethod toolkit based on existing frameworks which are summarized in Fig. 1 and Table 1. At its core is ecological momentary assessment (EMA)^24^, which captures physiological and psychological data in real-time within their natural context. We suggest complementing classical EMA by quantitative observations and non-real-time assessments of healthcare providers’ baseline psychophysiological characteristics. According to ISO 9241-210, user experience is influenced not only by system interaction but also by baseline psychophysiological factors, highlighting the importance of these non-real-time methods. Regarding these baseline factors, the principle of parsimony should guide the selection, focusing solely on individual factors that have either been shown to influence acute psychophysiological markers of interest in the general population^25–27^ or proven relevant specifically for healthcare providers, such as factors affecting general adherence to guidelines^28,29^, the adoption of AI, or general attitudes and beliefs about AI^30,31^.

**Table 1:** Real-world assessment frameworks that have informed the proposed toolkit

Conceptual Focus	Frameworks	Merits
User Experience	Model of User Experience^21^	Focus on how user experience is shaped by the balance between pragmatic quality (usability and functionality) and hedonic quality (pleasure, stimulation, and identity formation).
	Components of User Experience Model^23^	Focus on three key components of user experience (perception of instrumental and non-instrumental qualities and emotional user reactions) during direct system interaction.
	The Inputs and Outputs of UX^22^	Focus on the multidimensional, cumulative, and context-dependent nature of user experience.
Technology Engagement	Technology Acceptance Model Framework^42^	Focus on the impact of enduring individual characteristics, such as attitudes toward technology, perceived usefulness, and perceived ease of use on actual system use.
	Technostress Framework^43,44^	Focus on stress-inducing factors that affect well-being, performance, and emotional user responses during interactions with technology.
Psychophysiological State Evaluation	Component Model of Emotions^45^	Focus on core components of emotion-related psychophysiological states, including physiological response, cognitive appraisal, subjective experience, expressive behavior, and action tendencies.
	Ecological Momentary Assessment^24^	Focus on real-time capture of subjective experiences under real-world conditions, minimizing recall bias and maximizing ecological validity.
	Concept of additional non-metabolic changes in physiological markers indicating psychological states^46,47^	Focus on detection of relevant psychophysiological states through changes in physiological markers, which are unrelated to metabolic demands and do not require active involvement from the participant.
	AI-based automatic detection of psychophysiological states from video data^48–50^	Focus on the identification of relevant psychophysiological states through computer-based evaluation of video data.

Together, these methods would form a comprehensive toolkit that captures healthcare providers’ real-time and real-world experiences outside the laboratory. This approach minimizes the burden on clinicians by incorporating non-participatory observational and physiological measures. To ensure broad clinician acceptance, providing clinicians with thorough information on the purpose, data privacy management during data collection, and implementation of the toolkit is essential. Table 2 presents an overview of the tools and outcome variables included in our proposed approach.

**Table 2:** Tools and outcomes in real-world human-AI interaction assessment for healthcare providers

Time Frame	Tool	Outcomes
Real-time	Wearable sensors	Cardiovascular (heart rate, heart rate variability, blood pressure), respiratory (respiration, blood oxygen level), and autonomic measures (electrodermal activity, skin temperature); contextual information (body posture, motion)
	App-enabled devices	Psychophysiological (arousal, stress, exhaustion, relaxation), cognitive (cognitive effort, alertness), and affective (happiness, anxiety, sadness, calmness) states; other perceptions (perceived trust in the system/decision); contextual information (social and physical environment)
	(Video) quantitative observations	Behavioral indicators of psychological states, interaction and temporal (duration/frequency) patterns, contextual information (social and physical environment)
Non-real-time	Wearable sensors	Baseline physiological markers (cardiovascular, respiratory, autonomic)
	Questionnaires	Stable individual (age, gender/sex, BMI), workplace (years of experience; professional qualification), and digitalization-related (digital competence, AI-related attitudes, acceptance, and adherence) characteristics

Importantly, we suggest limiting the toolkit to a pragmatic set of quantitative and scalable instruments. The number of AI methods for healthcare is rapidly growing. Keeping up with this growth and providing quantitative evidence for human-AI interactions at scale requires instruments that are practical for large participant samples. In the realm of physiological assessment, this would favor the use of wearable consumer electronics, such as smart watches, over bespoke devices using professional diagnostic-grade electrodes due to lower cost, faster setup, and reduced disruption in real world environments. This would also favor the use of short, quantitative, validated items, pushed to participants during or after the interaction with AI systems, rather than semi-structured or structured one-on-one interviews. As an example, it would be impractical and even disruptive to require primary care physicians to wear electrocardiogram electrodes and be subjected to lengthy unstructured interviews after using an AI application aimed at better managing their patients. Rather, it would be clearly preferable to have them wear smartwatches and answer quick, structured questionnaires on their mobile devices during and after their use of AI applications in their daily practice. Only if we can easily scale psychophysiological measurements of human-AI interactions to hundreds or thousands of doctors will we be able to provide quantitative real-world evidence for healthcare AI.

The idea of integrating EMA into the investigation of human-AI interactions is not new. It has been discussed before, for instance by Chen et al.^32^. However, it has yet to be effectively implemented in human-AI interactions in healthcare. Where EMA has been applied, the focus has predominantly been on psychological self-report data, often neglecting physiological measures. This is surprising given that previous conceptualizations of EMA have emphasized the need for complementary assessment of both psychological and physiological data. Only by integrating these data sources can we accurately assess user experience, especially since self-reports are prone to biases and rely heavily on an individual’s ability to perceive or articulate their internal states. The interpretation of physiological measures requires careful integration with psychological measures: While physiological data can help to identify episodes that may be critical for user experience, psychological measures are essential for determining the underlying reason, as very different causes can exhibit similar arousal patterns in the markers of interest. Incorporating contextual information, such as motion and body position can further enhance this accuracy.

Preferably, the selection of specific markers should be guided by the respective aspect of experience that is of primary interest. Priority should be given to markers that have been frequently associated with these experiences and can be reliably measured using current technology. These include cardiovascular, respiratory, and autonomic measures. With regard to the choice of the right technology, sometimes also referred to as mobile Health (mHealth) devices, many consumer devices do not provide sufficient data quality or use proprietary, undisclosed algorithms for preprocessing physiological signals, and some raise concerns regarding data privacy. In contrast, devices offering high-quality raw physiological data that meet strict data protection standards are often expensive and lack the wearing comfort of consumer options, which can affect the willingness of healthcare professionals to participate and complicate large-scale data collection. Therefore, the selection of both markers and technologies should be carefully considered based on the specific questions and circumstances of the respective user experience evaluation.

The real-time collection of healthcare providers’ psychological data via self-rated items inevitably disrupts workflows to some degree but remains crucial for understanding user experience during human-AI interactions. The aim should be to comprehensively capture key aspects of psychological experience, including psychophysiological, cognitive, and affective dimensions, alongside other perceptions and contextual information as control variables. To minimize the burden on healthcare providers, we ideally propose embedding these assessments seamlessly within the respective AI system. For example, a radiologist using a decision support system could answer relevant items assessing endpoints such as usability, stress, workload, and diagnostic confidence directly within the system, without additional workflow disruptions. Should this not be feasible, following standard EMA procedures^24^, items can be delivered through app-enabled devices. Additionally, selecting only a minimal set of validated, hypothesis-driven items ensures that assessments remain focused and minimally intrusive.

While EMA is effective for capturing real-time data, fully structured observations offer additional objective insights without requiring active participation from healthcare providers, reducing their effort and integrating seamlessly into clinical workflows. Unlike unstructured observations, they categorize numerical data for predefined variables into standardized schemes. LM-based video analysis could support this by converting unstructured observational data into structured formats, addressing interrater reliability issues and enabling large-scale, reliable observations. A practical application example would be the use of computer cameras to record healthcare professionals during documentation in a Clinical Information System (CIS) and analyze changes in facial expressions, body posture, and movements to infer psychological states. Critically, data protection rights of both healthcare professionals and patients must always be respected.

## AI analyzing human-AI interaction

As we propose a scalable toolkit to evaluate human-AI interaction in real-world healthcare, we must consider: who will analyze the vast data generated? Observing hundreds of healthcare professionals using AI decision-support systems will produce an enormous dataset, including psychological and physiological measures. Unlike small-scale lab studies, real-world evaluations require automated approaches to process large-scale observational data.

To tackle this, we propose using AI to analyze the data. LLMs can already today make the analysis of clinical data much more efficient than a manual evaluation^33^. Also, AI can already interpret time series data obtained with wearable sensors^34^. Similarly, video-language models (VLMs) can assess recorded interactions, identifying events, but also human reactions to them. Ultimately, instead of relying on human evaluators, AI tools can perform sentiment analysis, behavior tracking, and physiological signal interpretation efficiently. AI-based analysis must be transparent, interpretable, and aligned with healthcare needs, but it will likely be the most efficient way to cope with the amount of measurement data when our proposed toolkit is deployed at scale.

## Regulation and human-AI interaction

AI-based methods are regulated according to medical device laws and guidance. Therefore, we have to take the regulatory landscape into account when we measure human-AI interaction. Relevant regulatory frameworks, include the Human Factors or Usability Engineering (ISO/IEC 62366-1:2015) framework. This has long had approaches for the quantitative evaluation of human-AI interactions with devices, systematizing the application of knowledge about human behavior, abilities, limitations, and other human characteristics into the design process, including software-driven user interfaces. These principles extend to the design of user documentation and user training to enhance safe and effective use. They recognize the close dependency between user experience and task performance, as safety issues with medical devices are often linked to user errors resulting from poor design and inadequate user experience. However, their focus is primarily on laboratory settings rather than real-world use of medical devices in healthcare, highlighting a weakness in medical device regulation^35^.

However, most principles and concepts for device development and evaluation predate the application of AI in medicine, and are therefore not tailored to AI. Often, these regulation frameworks are generic, and do not address the specifics of AI-enabled medical devices and their user interfaces. These frameworks were developed to be applicable to the wide variety of medical device types, some physical, some consisting of hardware and software, and some which are purely software. AI-enabled software brings new challenges in user experience engineering as it interacts in complex ways with human decision-making that did not apply in prior medical devices. Hence, in this way, AI-enabled decision-support tools act as human-AI interfaces, and therefore interfaces that influence cognitive and emotional states of medical decision-makers. It is therefore critical that these AI tools are developed in a manner that avoids automation bias, complacency bias and deskilling^9,35^.

These unique aspects of AI-enabled decision-support tools are highly important and safety and acceptable user experience (such as the avoidance of stress for workers) therefore warrants specific standards and guidance - which does not yet exist. Some standards and guidance are in preparation^36,37^ that begin to address some of these issues, but they do not explore newer challenges, such as the AI-enabled decision-support tools based on large language models that are entering regulatory approval pathways^38^, which have highly adaptive user interactions and experiences unlike any previous medical device^39^. Also, these draft standards are based on the experience of a small number of developers and the empirical knowledge of small expert groups rather than being based on real-world assessments. Overcoming these weaknesses requires a foundation from academic studies that map out and quantify human-AI interaction in the real world.

For these reasons, medical AI needs specifically designed frameworks for quantitatively evaluating real-world human-AI interactions which better take account of systemic interaction of healthcare providers with all AI contact points, instead of isolated focus on the design of individual devices. Under current frameworks the holistic experience of the human is neglected and much greater consideration of their mid- and long-term interaction with AI, and of their learning curve and adaptation to these technologies. Isolated studies have explored the human factors challenges of implementing AI systems, developing individual frameworks for human factors evaluation due to the absence of accepted standard approaches. In one such study, Google Health explored real-world challenges of an on-market deep learning algorithm for detecting diabetic retinopathy in an observational study in clinical environments in Thailand^40,41^. The authors identified a high image-quality related rejection rate of images that human readers could screen, with resultant increased staff workload, elevated stress, and longer patient waiting times. This is a typical example where user experience design, through better interaction between the system and user in required image properties, can increase performance and efficiency and reduce stress. The authors advocated for human-centered evaluative research to be conducted alongside prospective model accuracy evaluations.

## Conclusion and outlook

The value of medical AI lies in its interaction with human users, yet the lack of evaluation frameworks tailored to real-world environments remains a significant hurdle, because existing approaches fail to capture the complexity and contextual dependencies of clinical workflows in real-life settings. Here, we propose a multimodal toolkit which bridges the gap between laboratory research and clinical reality by integrating real-time physiological monitoring, observational data, and user experience assessment. In our concept, we focus on healthcare professionals as the users of AI systems. However, patients themselves are also interacting with AI systems and this interaction deserves substantial scientific attention in future studies. We intend to validate our proposed framework empirically in future field studies, and we encourage the scientific community to adopt and build on these concepts and likewise validate this approach across diverse healthcare settings to refine AI implementation further.

## Data Availability

No datasets were generated or analysed during the current study.
